# Corticosteroid Risk Function of Severe Infection in Primary Immune Thrombocytopenia Adults. A Nationwide Nested Case-Control Study

**DOI:** 10.1371/journal.pone.0142217

**Published:** 2015-11-11

**Authors:** Guillaume Moulis, Aurore Palmaro, Laurent Sailler, Maryse Lapeyre-Mestre

**Affiliations:** 1 UMR 1027, INSERM, Université de Toulouse III, Faculté de Médecine, Toulouse, France; 2 Service de Médecine Interne, Centre Hospitalier Universitaire de Toulouse, Toulouse, France; 3 CIC 1436, Centre Hospitalier Universitaire de Toulouse, Toulouse, France; 4 Service de Pharmacologie Médicale et Clinique, Centre Hospitalier Universitaire de Toulouse, Faculté de Médecine, Toulouse, France; Robert Bosch Hospital, GERMANY

## Abstract

Corticosteroid (CS)-related infection risk in immune thrombocytopenia (ITP) is unknown. The aim of this study was to assess the adjusted CS risk function of severe infection in persistent or chronic primary ITP adults. We designed a nested case-control study in the FAITH cohort. This cohort is built through the French national health insurance database named SNIIRAM and includes all treated incident persistent or chronic primary ITP adults in France (ENCePP n°4574). Patients who entered the FAITH cohort between 2009 and 2012 were eligible (n = 1805). Cases were patients with infection as primary diagnosis code during hospitalization. Index date was the date of first hospitalization for infection. A 2:1 matching was performed on age and entry date in the cohort. Various CS exposure time-windows were defined: current user, exposure during the 1/3/6 months preceding index date and from the entry date. CS doses were converted in prednisone equivalent (PEQ). The cumulative CS doses were averaged in each time-window to obtain daily PEQ dosages. Each CS exposure definition was assessed using multivariate conditional regression models. During the study period, 161 cases (9 opportunistic) occurred. The model with the best goodness of fit was CS exposure during the month before the index date (OR: 2.48, 95% CI: 1.61–3.83). The dose-effect relation showed that the risk existed from averaged daily doses ≥5 mg PEQ (vs. <5 mg: 2.09, 95% CI: 1.17–3.71). The risk of infection was mainly supported by current or recent exposure to CS, even with low doses.

## Introduction

Immune thrombocytopenia (ITP) is a bleeding disorder due to an autoimmune reaction directed against platelets and megakaryocytes [1,2]. It is referred to as “primary” when not associated to another disease (about 80% of ITPs) [1]. First-line treatment is based on corticosteroids [3,4]. In case of severe bleeding, intravenous polyvalent immunoglobulin (IVIgs) is added [5]. In adults, 70% of ITPs become persistent (lasting ≥3 months) or chronic (lasting ≥12 months) [1,6–8]. Nowadays, several corticosteroid-sparing treatments targeting the immune system are available [3,4]. Splenectomy is the reference treatment for corticosteroid dependent or resistant ITP [4]. Rituximab, a monoclonal antibody directed against CD20 has been used since the 2000s. It was the leading non-corticosteroid treatment used during the persistency phase in France between 2009 and 2011 [9]. Immunosuppressants such as azathioprine, mycophenolate or cyclosporine are less frequently used [3,9]. Lastly, IVIg is not recommended as chronic treatment [3,4]. Nevertheless they are widely used in some countries [9].

However, these second-line treatments may take time to be effective. During this time, corticosteroids-dependent patients are still exposed to corticosteroids, which are secondarily tapered. Consequently, adult ITP adults may be persistently exposed to low dose corticosteroids during the initial management of ITP.

An increased risk of infection has been demonstrated in ITP patients [10–13]. The hazard ratio for 20-year mortality caused by infection in adult ITP patients has been estimated to 2.4 (95% confidence interval -95% CI: 1.0–5.7) as compared with the general population [14]. A study carried out in the General Practice Research Datalink patients between 1992 and 2005 with 1145 ITP showed that 19% of deaths were related to infection [15].

One might suppose that this high risk of infection in ITP is related to the exposure to immunosuppressive treatments or splenectomy [12,16–18]. Surprisingly, the risk associated with corticosteroid use [19] has not been assessed in ITP, albeit corticosteroids have been the cornerstone treatment of this disease for 60 years [20]. The infectious risk of splenectomy has never been adjusted on corticosteroid exposure [16–18]. In daily practice, rituximab is often used in patients with contra-indication for splenectomy [9,21]. The risk of infection on rituximab was reassuring in the sole phase II clinical trial [22], however some reports of rituximab use in “real-life” practice raised concerns about the risk of infection [23–25]. Once again, most of the patients who experienced severe infection were also exposed to corticosteroids.

The aim of this study was to assess the risk function of corticosteroid-related severe infection in persistent or chronic primary ITP adults, adjusted for splenectomy, rituximab, other immunosuppressant and intravenous immunoglobulin (IVIg) exposures.

## Materials and Methods

We performed a nested case-control study in the so-called FAITH (French Adult Immune Thrombocytopenia: a French pHarmacoepidemiological study) cohort. This study is registered in the post-authorization survey registry of the European Network Centers for Pharmacoepidemiology and Pharmacovigilance (ENCePP) coordinated by the European Medicine Agency (study n°4574). Full protocol of the FAITH study has been described elsewhere [26]. STROBE checklist is indicated in S1 File.

### Data Source

The source of data was the French health insurance database, named *Système National d’Information Inter-Régimes de l’Assurance Maladie* (SNIIRAM) [27]. This database contains individualized, anonymous and linkable data. These data are prospectively recorded for every patient benefitting from health care in France, thus virtually covering the entire French population (66 million inhabitants). They include data regarding demographics, long-term disabling diseases (LTD) allowing full medical expenditure reimbursement, hospitalizations, out-hospital procedures and drug dispensing (dates of dispensing, drug identifiers and numbers of units dispensed). Hospitalizations data include procedures, diagnosis codes (one principal, one related and up to 30 associated diagnoses encoded with the International Classification of Disease, version 10 –ICD-10) and costly drugs dispensed such as rituximab or IVIg. Data regarding out- and in-hospital drug dispensing include the drug name, the dosage and the form, as well as the quantity delivered and the date of dispensing [27].

### Population source

The patients’ selection process has been described in details elsewhere [26]. Briefly, data of all the patients with at least one ITP code as LTD or during hospital stays (D69.3 code of the ICD-10) were extracted from the SNIIRAM between 2009 and 2012. The date of diagnosis was defined as the first diagnosis code or the first dispensing of ITP drug before the first diagnosis code, if any. After excluding patients with ambiguous or contradictory codes suggesting miscoding, and after exclusion of secondary ITP, we restricted the cohort to incident and persistently treated patients [26]. Persistent treatment was defined by the exposure to any ITP treatments exceeding three consecutive months (corticosteroids, IVIg, dapsone, danazol, thrombopoietin receptor agonists or other immunosuppressants), to splenectomy or to rituximab. Follow-up started at the first exposure to persistent treatment (entry date in the cohort) [26]. For the present study, we analyzed the sample of patients included from the 1^st^ July 2009 to the 30^th^ June 2012.

### Case-control design

Cases were patients who experienced a severe infection after the entry date in the cohort. Severe infection was defined by an in-hospital primary diagnosis with an ICD-10 code for infection (Table A in S2 File). This list of codes of infection as primary diagnosis has a predictive positive value of 97% (95% CI: 93–100%) in the SNIIRAM data (personal data). Cases’ index date was the date of first severe infection. Controls were randomly selected among the patients without any severe infection during the follow-up and followed until the corresponding case’s index date.

Two controls were matched to each case on the age at entry date in the cohort (<65 vs. ≥65 years), the year and the month of entry date in the cohort. This latter matching condition was used to control for seasonality and epidemics. Controls’ index dates were index dates of the corresponding cases. Consequently, disease duration from entry date to index date was similar for cases and controls.

### Corticosteroid exposure

For corticosteroids, we considered various time-windows: exposure at index date, exposure during 1, 3 or 6 months before the index date (defined as ever exposed during the 1/3/6 months before the index date) and any exposure from the entry date to the index date. Dispensed doses were converted in prednisone equivalent (PEQ). To assess the effect of cumulative exposure, the cumulative doses received during the quoted time-windows were averaged for each patient to obtain daily PEQ dosages.

### Potential confounders

We identified splenectomy before index date through hospital procedures codes. We considered that a patient was exposed to rituximab during the six months following infusion, thus it is the period of maximal B-cell depletion with the maximal risk of infection [28]. However, we also carried out sensitivity analyses considering the 3-month and the 12-month periods after rituximab, as well as the “ever exposed” definition. For azathioprine, mycophenolate, cyclosporine and IVIg, we searched exposure during the month before the index date. We also carried out sensitivity analyses using 3-, 6-, 12-month periods and the “ever exposed” definition.

Other covariates were gender, mucosal or internal bleeding at ITP onset (reflecting disease severity) [8] and comorbidities increasing the risk of infection: diabetes mellitus, chronic cardiac, lung, kidney and liver diseases (considered separately). Comorbidities were identified using algorithms validated in the SNIIRAM [29]. They combined LTD, in-hospital diagnosis codes, specific procedures (*i*.*e*. hemodialysis for severe chronic disease) and specific drugs dispensing (*i*.*e*. glucose lowering drugs for diabetes mellitus). Of note, patients with cancer, connective tissue disease, chronic viral infection and primary immune deficiency were considered as secondary ITP patients and were not included in the FAITH cohort [8,26].

### Statistical Analysis

We performed a model of conditional logistic regression for each corticosteroid exposure time-window. Models’ goodness of fit was compared using the Akaike criterion [30]. We also compared the corticosteroid exposure time-windows in specific models so as to compare the effect of recent *versus* past exposure. Lastly, we explore the dose-effect relationship in the best model. All the models were adjusted for the potential confounders quoted above.

Non-costly drug dispensing during hospitalization stays is not recorded in the SNIIRAM. Therefore, we carried out sensitivity analyses adding in the models a variable corresponding to the occurrence of a hospitalization of at least 7 days between start of follow-up and index date, *i*.*e*. hospitalization at risk for a significant unmeasured corticosteroid exposure.

Calculations were carried out using SAS 9.4^™^ statistical software (SAS Institute, Cary, North Carolina, USA).

### Ethical considerations

This study received all mandatory authorizations according to French law (*Institut des Données de Santé* approval in March 2012, numbered 40; and *Commission Nationale de l’Informatique et des Libertés* authorization in July 2012, decision numbered DE-2012-076 regarding the request numbered 1579257).

## Results

### Selection of the population

During the study period, 1805 incident primary ITP adults persistently treated entered the FAITH cohort. Mean age was 57.6 ± 21.3 years with 58.9% of females. Mean follow-up was 18.5 ± 6.8 months. One hundred and sixty-four patients (9.1% of all FAITH patients) had a severe infection and were matched to 321 controls. For three cases (2 gastro-intestinal infections and 1 pneumonia), no suitable match could be identified. All other cases had 2 controls except one case (one control only). Therefore, 482 patients were included the case-control study (161 cases and 321 controls).

### Characteristics of cases and controls

The main characteristics of cases and controls are detailed in Table 1. Mean disease duration from entry date until index date (date of severe infection) was 8.7 ± 6.1 months. Cases were more frequently males (57.1% vs. 44.2%, p = 0.007) and had more comorbidities. Cases were more frequently exposed to splenectomy, rituximab, immunosuppressants (azathioprine, mycophenolate or cyclosporine) and IVIg in the various time-windows, albeit not reaching statistical significance (Table 1). Nine patients were exposed to repeated courses of rituximab before index date (including 6 cases and 3 controls).

**Table 1:** Cases’ and controls’ characteristics.

Characteristics	Cases (n = 161)	Controls (n = 321)	*p*
Age at T0*, years, mean ± SD	69.0 ± 18.6	67.4 ± 18.3	matching
Females, n (%)	69 (42.9%)	179 (55.8%)	0.007
Mucosal bleeding at diagnosis, n (%)	13 (8.1%)	18 (5.6%)	0.30
Cardiac disease, n (%)	15 (9.3%)	21 (6.5%)	0.27
Chronic pulmonary disease, n (%)	17 (10.5%)	20 (6.2%)	0.092
Chronic kidney disease, n (%)	13 (8.1%)	9 (2.8%)	0.009
Chronic liver disease, n (%)	0	0	-
Diabetes mellitus, n (%)	15 (9.3%)	37 (11.5%)	0.46
Disease duration from T0* until the index date^†^, months, mean ± SD	8.7 ± 6.1	8.7 ± 6.1	matching
Exposure to splenectomy before the index date^†^, n (%)	10 (6.2%)	17 (5.3%)	0.72
Exposure to rituximab			
In the three months before the index date^†^, n (%)	20 (12.4%)	27 (8.4%)	0.161
In the six months before the index date^†^, n (%)	28 (17.4%)	39 (12.1%)	0.127
In the year before the index date^†^, n (%)	34 (21.1%)	69 (21.5%)	0.92
At any time from T0* until the index date, n (%)	41 (25.5%)	89 (27.5%)	0.60
Exposure to azathioprine, mycophenolate or cyclosporine			
In the month before the index date ^†^, n (%)	5 (3.1%)	4 (1.2%)	0.169
In the three months before the index date^†^, n (%)	7 (4.3%)	8 (2.5%)	0.27
In the six months before the index date^†^, n (%)	7 (4.3%)	8 (2.5%)	0.27
At any time from T0* until the index date, n (%)	9 (5.6%)	8 (2.5%)	0.082
Exposure to intravenous immunoglobulin			
In the month before the date^†^, n (%)	22 (13.7%)	30 (9.3%)	0.149
In the three months the before index date^†^, n (%)	36 (22.6%)	55 (17.1%)	0.167
In the six months the before index date^†^, n (%)	47 (29.2%)	81 (25.2%)	0.35
At any time from T0* until the index date, n (%)	78 (48.4%)	152 (47.3%)	0.82

*T0: entry date in the FAITH cohort (date of start of persistent treatment for ITP).

^†^Index date: date of infection for cases.

Abbreviations: SD: standard *deviation*.

### Infections

Infections are categorized in Table 2. Localizations accounting for more than 10% of infections were the lower respiratory (33.5%), the gastro-intestinal (16.8%), and the urinary tracts (13.0%) as well as the skin (12.4%). Ten patients presented opportunistic infections: 3 zosters, 3 apergilloses, 2 pneumocytoses, 2 tuberculoses and 1 varicella. Opportunistic infections that occurred are detailed in Table B in S2 File. No Progressive multifocal leukoencephalopathy was described.

**Table 2:** Description of severe infections that occurred during the follow-up of the 1805 incident primary ITP adults persistently treated who entered the FAITH cohort between the 1^st^ of July 2009 and the 30^th^ of June 2012.

Types of infection	n (%)
***By site***	
Pulmonary	54 (33.5%)
Gastro-intestinal tract	27 (16.8%)
Uro-genital tract	21 (13.0%)
Cutaneous	20 (12.4%)
Sepsis	11 (6.8%)
ENT/ocular	8 (5.0%)
Neurological	2 (1.2%)
Pyogenic arthritis	2 (1.2%)
Endocarditis	1 (0.6%)
***By microorganism***	
Gram-negative bacillus	7 (4.3%)
*Streptococcus pneumoniae*	5 (3.1%)
Varicella-zoster virus	4 (2.5%)
Influenza	3 (1.7%)
Aspergillosis	3 (1.7%)
Tuberculosis	2 (1.2%)
Pneumocystosis	2 (1.2%)
*Staphylococcus*	2 (1.2%)
Cytomegalovirus	2 (1.2%)
*Haemophilus influenzae*	1 (0.6%)
*Candida*	1 (0.6%)
Parvovirus	1 (0.6%)
Leishmaniosis	1 (0.6%)

### Corticosteroid risk function of infection

Results of the univariate analyses are shown in Table C in S2 File. Results of the multivariate models are described in Table 3. Considering the various time-windows of exposure to corticosteroids in multivariate models, the Akaike criterion was the lowest for exposure during the month before the index date (adjusted odds ratio: 2.48, 95% CI: 1.61–3.83). For longer time-windows, the risk decreased nevertheless remained significant until the six-month time-window. When comparing the time-windows, there was no statistically significant increased risk among the patients exposed during the month before the index date whether current user or not at index date (adjusted odds ratio: 1.23, 95% CI: [0.63–2.40]). In contrast, there was a major risk of infection among the patients ever exposed during the 3 months prior to the index date when exposed during the month before the index date in comparison with those not exposed during the month before the index date (adjusted odds ratio: 3.42; 95% CI: [1.31–8.92]).

**Table 3:** Multivariate models* assessing the link between exposure to corticosteroids during the various time-windows and occurrence of severe infection. Each line shows the adjusted result of exposure to corticosteroids with a given time-window period.

Exposure to corticosteroids	OR [95% CI]	*p*	*Akaike criteria*
***Time-window periods***			
Exposure to corticosteroids at index date^†^	2.12 [1.39–3.23]	<0.001	337.553
Exposure to corticosteroids in the month before the index date^†^	2.48 [1.61–3.83]	<0.001	331.921
Exposure to corticosteroids in the 3 months before the index date^†^	1.96 [1.25–3.09]	0.003	341.189
Exposure to corticosteroids in the 6 months before the index date^†^	1.64 [1.01–2.64]	0.044	342.859
Exposure to corticosteroids from the cohort entry date until the index date^†^	1.22 [0.65–2.29]	0.53	346.674
***Some comparisons across time-window periods***			
Among patients with exposure in the month before the index date (n = 255, incl. 105 cases): current user vs. not current user at index date ^†^	1.23 [0.63–2.40]	0.55	133.718
Among patients with exposure in the 3 months before the index date^†^ (n = 300, incl. 113 cases): exposure in the month the before index date vs. no exposure in the month before the index date^†^	3.42 [1.31–8.92]	0.012	161.080
Among patients with exposure in the 6 months before the index date (n = 345, incl. 124 cases): exposure in the 3 months before the index date^†^ vs. no exposure in the 3 months before the index date^†^	2.57 [0.95–7.00]	0.064	195.633

*Adjusted for mucosal or internal bleeding at diagnosis, lung disease, kidney disease, cardiac disease, diabetes mellitus, exposure to rituximab in the six months before index date, to azathioprine, mycophenolate, cyclosporine and polyvalent immunoglobulin in the month before index date as well as splenectomy before index date. Age and disease duration until index date were neutralized by matching.

^†^Index date: date of infection for cases.

Abbreviations: 95% CI: 95% confidence interval; OR: odds ratio.

Then, we assessed the corticosteroid dose-effect relationship in the best model among the models previously quoted, considering corticosteroid exposure during the month before the index date. The analyses were also adjusted for corticosteroid past cumulative dose during the five months preceding the month before the index date. We identified a statistically significant association from an average daily dose ≥5 mg PEQ/day during the month before the index date (adjusted odds ratio: 2.09, 95% CI [1.17–3.71], p = 0.01). We did not find any impact of corticosteroid cumulative dose during the five months preceding the month before the index date (Table 4), as well as for corticosteroid cumulative dose from the entry date in the cohort until the month before the index date (sensitivity analysis, not shown).

**Table 4:** Multivariate model* assessing the dose-effect relation between exposure to corticosteroids during the month before the index date and occurrence of severe infection.

Average daily dose of corticosteroids in the month before the index date ^†^, mg PEQ	OR [95% CI]	*p*
Male gender	1.63 [1.11–2.40]	0.01
Average daily dose of corticosteroids in the month before the index date ^†^, mg PEQ		0.003
<5	1	-
[5–10[	2.09 [1.17–3.71]	0.01
≥10	1.99 [1.26–3.17]	0.003

*Adjusted for gender, mucosal or internal bleeding at diagnosis, lung disease, kidney disease, cardiac disease, diabetes mellitus, exposure to rituximab in the six months before index date, to azathioprine, mycophenolate, cyclosporine and polyvalent immunoglobulin in the month before index date, splenectomy before index date, as well as cumulative exposure to corticosteroids during the five months preceding the month before the index date. Age and disease duration until index date were neutralized by matching. Akaike criteria: 340.154.

^†^Index date: date of infection for cases.

Abbreviations: 95% CI: 95% confidence interval; OR: odds ratio.

Sensitivity analyses using the 3-, 6-, 12-month periods and the “ever exposed” definition for splenectomy, IVIg and immunosuppressants, as well as using the 3-month period, the 12-month period and the “ever exposed” definition for rituximab led to similar results (data not shown).

Of note, male gender was associated to severe infection in all multivariate models, with odds ratio ranging from 1.55 to 1.86. It was the sole covariate associated with severe infection in the final multivariate model (Table D in S2 File). Exposure to rituximab, immunosupressants and IVIg as well as splenectomy could be removed from all multivariate models. However, rituximab exposure during the six months before index date was near to the significance threshold in the final multivariate model (adjusted odds ratio: 1.67, 95% CI [0.96–2.90], p<0.07; see Table E in S2 File).

Sensitivity analysis adding in the models the occurrence of hospitalization of at least 7 days led to similar results. However, this variable was independently associated with infection occurrence (Table F in S2 File).

## Discussion

This study demonstrated that corticosteroid exposure was a leading factor associated with the occurrence of severe infection in incident primary ITP adults persistently treated. The risk function showed a maximal risk period in the month preceding the date of risk measure. The risk was present even in patients exposed to low, supraphysiologic daily doses (≥5mg PEQ/day). This is an important finding as many physicians may have in mind that the infectious risk with corticosteroids is for short exposures above 20 mg PEQ daily, as noted in previous reports [31,32]. In contrast, past exposure prior to the month preceding the date of risk measure and past cumulative dose does not seem to play a major role in the occurrence of severe infection.

This risk function is similar to the one recently reported in older rheumatoid arthritis patients [33]. The increased risk with recent exposure might be explained by the effect of corticosteroids on innate immunity, which is rapidly reversed when the drug is stopped [19]. The increased risk for supra-physiological (≥5mg PEQ/day) exposure to corticosteroids is an argument to support this finding in a pathophysiological perspective. A similar dose-effect relationship has been demonstrated in rheumatoid arthritis after adjustment on other immunosuppressive treatments [33,34].

Importantly, 130 patients (27.0%) were exposed to rituximab during the study period, of whom 67 (13.9%) were exposed during the six months before index date. In the univariate analysis, there was a clear trend towards an increased risk of infection in the 6 months following rituximab (odds ratio: 1.51, 95% CI [0.90–2.56], p = 0.1). This trend was confirmed in the multivariate analysis (adjusted odds ratio: 1.67, 95% CI [0.96–2.90], p<0.07). Consequently, we cannot exclude an increased risk with rituximab due to sample size. In the previously quoted French registry stemming from referral centers, 11 infections occurred among 248 ITP patients treated with rituximab (median time to infection: 4 months; range: 1–14 months). Among them, 7 patients were also exposed to corticosteroids at the time of infection [25]. Further larger population-based studies are needed to assess the adjusted rituximab risk function of infection. Similarly, the respective weight of splenectomy and exposure to immunosuppressants (azathioprine, mycophenolate and cyclosporine) could not be assessed in the multivariate models due to the few number of patients exposed to either treatment before the index date (respectively, n = 27 and n = 19).

Our study points the role of gender in the risk of infection. We have no clear explanation for this latter association. Unfortunately, detailed clinical data such as tobacco exposure are not recorded in the SNIIRAM. Cases had more comorbidities than controls, particularly of chronic pulmonary and chronic kidney disease. Chronic pulmonary disease may favors lung infections (accounting for one third of the infections observed in this study), and chronic kidney disease has been demonstrated as a risk factor for infection [35,36].

The pattern of severe infection sites was consistent with data from the Danish population-based study [11]. Of note, 9/161 (5.6%) of the severe infections we observed were opportunistic. The risk of varicella-zoster, parasitic (n = 5 in our study) and fungal infections has been reported in ITP patients [37–42]. It is explained by the effect of corticosteroids on innate immunity (for instance as regards the cases of aspergillosis) and on T-lymphocytes [19].

In sensitivity analyses adding in the models a variable corresponding to the occurrence of a hospitalization of at least 7 days between start of follow-up and index date, this variable was independently associated with severe infection occurrence. Three explanations may be suggested: i) hospitalization reflects a more severe ITP, or in patients with comorbidities with a higher baseline risk for infection, ii) hospitalization in itself may increase the risk of infection and iii) this association is due to unmeasured exposure to corticosteroids.

Some limitations should be discussed. As all studies conducted in health insurance databases, the identification of ITP patients relies on diagnosis codes. Possibility of miscoding cannot be fully excluded despite all cautions [8,26]. The current study includes persistently treated primary ITP adult patients. Thus, our results cannot be extended to all ITP patients. The codes of infections corresponded mostly to infection localization rather than to the microorganism in cause. Consequently, we could not stratify the analyses on the type of microorganism. We could not exclude that the risk may be different between bacterial, viral and parasitic infections. The nested case-control design was used to assess the corticosteroids risk function of severe infection. The effect of repeated rituximab courses and of rituximab after or before splenectomy has to be investigated in the long term. Cases and controls were matched on the age and on disease duration. Consequently, we could not assess the weights of these factors that have been demonstrated as linked to hospital stay or visit for infection in a previous population-based study in ITP [11]. Out of the 1805 patients included in the FAITH cohort, 28 died during follow-up at home without any hospitalization for infection. As the cause of death is not recorded in the SNIIRAM, we cannot rule out that these patients died due to infection without being considered as cases. Similarly, the lack of clinical data available in the SNIIRAM prevented us from adjusting on some disease characteristics such as detailed bleeding score to assess disease severity [5] or tobacco exposure. Examination and lab tests results are not recorded in the SNIIRAM. Consequently, we could not adjust the analysis on platelet count, that may be associated with the risk of infection [43–45]. We did not find any protective effect of IVIg, but a channeling bias (treatment with IVIg of patients with a higher baseline risk of infection) cannot be ruled out. Lastly, we used dispensing data to model drug exposure, and compliance could not be assessed.

## Conclusions

This study suggests that corticosteroids were the main immunosuppressant associated with severe infection in primary ITP adults exposed to persistent treatment. Maintaining corticosteroid even at a supra-physiological dose is associated with severe infection. In contrast, the past cumulative dose does not seem to play a major role. These results sustain the use of corticosteroid-sparing agents in persistent or chronic ITP.

## Supporting Information

S1 File STROBE Checklist. (DOC)

S2 File Supplementary Tables. **Table A.** International Classification of Diseases, version 10 (ICD-10) codes used for the identification of severe infections during hospital stays.
**Table B.** Description of opportunistic infections that occurred during the follow-up of the 1805 incident primary ITP adults persistently treated who entered the FAITH cohort between the 1^st^ of July 2009 and the 30^th^ of June 2012.
**Table C.** Univariate models assessing the link between exposure to corticosteroids during the various time-windows and covariates with the occurrence of severe infection.
**Table D.** Full multivariate model assessing the link between exposure to corticosteroids during the month before index date and occurrence of severe infection. This model had the lowest Akaike criterion value (331.921).
**Table E.** Full multivariate model assessing the link between exposure to corticosteroids during the month before index date and occurrence of severe infection, without withdrawal from the model of the exposure to rituximab in the month before index date.
**Table F.** Full multivariate model assessing the link between exposure to corticosteroids during the month before index date and occurrence of severe infection: sensitivity analysis adding the variable corresponding to the occurrence of a hospitalization of at least 7 days between start of follow-up and index date.
(DOCX)
