# Supplementary material for: Corticosteroid Risk Function of Severe Infection in Primary Immune Thrombocytopenia Adults. A Nationwide Nested Case-Control Study
Source: PLoS One. 2015 Nov 11;10(11):e0142217. doi: 10.1371/journal.pone.0142217 (PMC4641733; doi:10.1371/journal.pone.0142217)
Supplement: S2 File — Table A. International Classification of Diseases, version 10 (ICD-10) codes used for the identification of severe infections during hospital stays. Table B. Description of opportunistic infections that occurred during the follow-up of the 1805 incident primary ITP adults persistently treated who entered the FAITH cohort between the 1st of July 2009 and the 30th of June 2012. Table C. Univariate models assessing the link between exposure to corticosteroids during the various time-windows and covariates with the occurrence of severe infection. Table D. Full multivariate model assessing the link between exposure to corticosteroids during the month before index date and occurrence of severe infection. This model had the lowest Akaike criterion value (331.921). Table E. Full multivariate model assessing the link between exposure to corticosteroids during the month before index date and occurrence of severe infection, without withdrawal from the model of the exposure to rituximab in the month before index date. Table F. Full multivariate model assessing the link between exposure to corticosteroids during the month before index date and occurrence of severe infection: sensitivity analysis adding the variable corresponding to the occurrence of a hospitalization of at least 7 days between start of follow-up and index date. (DOCX) [file pone.0142217.s002.docx]

**Table A.** International Classification of Diseases, version 10 (ICD-10) codes used for the identification of severe infections during hospital stays.

| **ICD-10 label** | **ICD-10 codes** |
| --- | --- |
| Certains infectious diseases | A00-B99 |
| Nervous system infections | G00-G07, G53.0, G53.1, G63.0, G73.4, G94.0 |
| Ophtalmological infections | H00, H05.0, H06.1, H10.0, H13.0, H13.1, H19.0-H19.2, H22.0, H32.0, H44.0, H44.1, H45.1, H58.8, H59.8 |
| Eear, Nose, Throat infections | H60.0-H60.3, H62.0-H62.4, H66.0-H66.4, H67.0, H67.1, H70, H75.0, H94.0, J32, J34.0, J36, J37.8, J39.0, J39.1, K04.0, K04.1, K04.4, K04.6, K04.7, K05.2, K11.3, K11.2 |
| Cardiovascular system infections | I30.1, I32.0, I32.1, I33, I38, I40.0, I41.0-I41.2, I98.0, I98.1 |
| Upper respiratory tract | J00-J06 |
| Lung infections | J09-J18, J20-J22, J85.0-J85.2, J86 |
| Gastro-intestinal infections | D73.3, K23.0, K23.1, K35-K37, K57, K61, K63.0, K63.1, K65, K67, K75.0, K77.0, K80.0, K80.1, K80.3, K81, K87.1, K93.0, K93.1 |
| Dermatological infections | L00-L08 |
| Musculoskelettal system infections | M00, M01, M60.0, M63.0-M63.2, M65.0, M65.1, M68.0, M71.0, M71.1, M73.0, M73.1, M86, M90.0-M90.2 |
| Urinary and gynecologic tract infections | N08.0, N10, N12, N13.6, N15.1, N16.0, N22.0, N29.0, N29.1, N30.0, N33, N34, N39.0, N41, N43.1, N45, N48.1, N48.2, N49, N51, N61, N70-N76, N77.0, N77.1 |
| Infections during pregnancy (only for the mother) | O23, O75.3, O85, O86, O91, O98 |
| Others | U80, U81, U88, U89 |

**Table B.** Description of opportunistic infections that occurred during the follow-up of the 1805 incident primary ITP adults persistently treated who entered the FAITH cohort between the 1^st^ of July 2009 and the 30^th^ of June 2012.

| **Infection** | | **Site** | **Age** | **Gender** | **Comorbidities** | **Mucosal or internal bleeding at diagnosis** | **Time from entry in the cohort* to infection (months)** | **Drug exposure** |  |
| --- | --- | --- | --- | --- | --- | --- | --- | --- | --- |
| ***Pneumocystis*** | |  |  |  |  |  |  |  |  |
|  | **1** | - | 26 | Male | Chronic kidney disease | No | 12 | - CS (current user, for 12 months) - CsA (current user, for 7 months) |  |
|  | **2** | - | 66 | Female | Stroke | Yes | 4 | - Splenectomy 4 months ago - RTX 2.5 months ago |  |
| ***Tuberculosis*** | |  |  |  |  |  |  |  |  |
|  | **1** | Miliary | 72 | Female | No | Yes | 6 | - RTX 5 months ago |  |
|  | **2** | Ocular | 79 | Male | No | No | 17 | - CS past user (during 1 year, stopped 6 months ago) |  |
| ***Aspergillosis*** | |  |  |  |  |  |  |  |  |
|  | **1** | Not specified | 65 | Male | No | No | 21 | - CS past user (during 6 months, stopped 5 months ago) - RTX 1 month ago |  |
|  | **2** | Pulmonary | 71 | Male | No | No | 13 | - CS past user (during 11 months, stopped 6 months ago) - RTX 4 months ago |  |
|  | **3** | Pulmonary, invasive | 69 | Male | Lung disease | No | 6.5 | - CS (current user, for 7 months) |  |
| ***VZV*** | |  |  |  |  |  |  |  |  |
|  | **1** | Zoster | 75 | Male | Ischemic cardiac disease | No | 1.5 | - CS user during the month before infection but not current user, for 1.5 months) |  |
|  | **2** | Zoster | 85 | Male | No | No | 5 | - Splenectomy 3 months ago |  |
|  | **3** | Varicella | 82 | Female | No | No | 8 | - CS (current user, for 10 months) |  |
|  | **4** | Zoster | 73 | Male | Ischemic cardiac disease | No | 5 | - CS (current user, for 6 months) |  |

*Entry date in the FAITH cohort: date of start of persistent treatment for immune thrombocytopenia.

Abbreviations: CS: corticosteroids; CsA: cyclosporine; PEQ: prednisone equivalent; RTX: rituximab; VZV: varicella-zoster virus.

**Table C.** Univariate models assessing the link between exposure to corticosteroids during the various time-windows and covariates with the occurrence of severe infection.

| **Characteristics** | | | **OR [95% CI]** | ***p*** |
| --- | --- | --- | --- | --- |
| **Males** | | | 1.64 [1.12-2.39] | 0.010 |
| **Mucosal bleeding at diagnosis,** | | | 1.46 [0.71-3.03] | 0.30 |
| **Cardiac disease** | | | 1.48 [0.73-2.97] | 0.27 |
| **Lung disease** | | | 1.76 [0.90-3.44] | 0.098 |
| **Kidney disease** | | | 3.09 [1.27-7.49] | 0.013 |
| **Diabetes mellitus** | | | 0.78 [0.42-1.48] | 0.44 |
| **Exposure to corticosteroids** | | |  |  |
|  | Current user at index date^†^ | | 1.98 [1.31-2.97] | <0.001 |
|  | In the month before index date^†^ | | 2.34 [1.53-3.56] | <0.001 |
|  | In the three months before index date^†^ | | 1.85 [1.19-2.89] | <0.001 |
|  | In the six months before index date^†^ | | 1.63 [1.01-2.61] | 0.044 |
|  | At any time from T0* until index date | | 1.25 [0.67-2.33] | 0.48 |
|  | Among patients with exposure in the month before index date^†^ (n=255): current user vs. not current user at index date^†^ | | 1.16 [0.60-2.22] | 0.66 |
|  | Among patients with exposure in the 3 months before index date^†^ (n=300): exposure in the month before index date^†^ vs. no exposure in the month before index date^†^ | | 3.20 [1.28-8.00] | 0.013 |
|  | Among patients with exposure in the 6 months before index date^†^ (n=345): exposure in the 3 months before index date^†^ vs. no exposure in the 3 months before index date^†^ | | 2.44 [0.97-6.15] | 0.059 |
|  | Among patients with exposure from T0* to index date^†^ (n=427): exposure in the 6 months before index date^†^ vs. no exposure in the 6 months before index date^†^ | | 1.97 [1.02-3.79] | 0.042 |
|  | Daily dose at index date^†^, mg PEQ | |  | 0.001 |
|  |  | <5 | 1 | - |
|  |  | [5-10[ | 1.20 [0.55-2.60] | 0.64 |
|  |  | [10-20[ | 3.21 [1.43-7.17] | 0.004 |
|  |  | [20-30[ | 3.12 [1.48-6.56] | 0.003 |
|  |  | ≥30 | 2.26 [1.26-4.04] | 0.006 |
|  | Averaged daily dosage in the month before index date^†^, mg PEQ | |  | 0.002 |
|  |  | <5 | 1 | - |
|  |  | [5-10[ | 2.08 [1.17-3.68] | 0.012 |
|  |  | ≥10 | 2.01 [1.27-3.18] | 0.003 |
|  | Averaged daily dosage in the 3 months before index date^†^, mg PEQ | |  | 0.066 |
|  |  | <5 | 1 | - |
|  |  | [5-10[ | 1.16 [0.61-2.18] | 0.65 |
|  |  | ≥10 | 1.66 [1.08-2.56] | 0.020 |
|  | Averaged daily dosage in the 6 months before index date^†^, mg PEQ | |  | 0.058 |
|  |  | <5 | 1 | - |
|  |  | [5-10[ | 1.42 [0.75-2.68] | 0.28 |
|  |  | ≥10 | 1.72 [1.09-2.70] | 0.019 |
|  | Averaged daily dosage from T0* until index date^†^, mg PEQ | |  | 0.110 |
|  |  | <5 | 1 | - |
|  |  | [5-10[ | 1.11 [0.59-2.10] | 0.75 |
|  |  | ≥10 | 1.62 [1.00-2.63] | 0.052 |
| **Splenectomy before index date^†^** | | | 1.22 [0.51-2.93] | 0.61 |
| **Exposure to rituximab in the six months before index date^†^** | | | 1.51 [0.90-2.56] | 0.120 |
| **Exposure to immunosuppressant in the month before index date ^†^** | | | 2.50 [0.67-9.31] | 0.172 |
| **Exposure to intravenous immunoglobulin in the month before index date^†^** | | | 1.66 [0.87-3.17] | 0.121 |

* T0: entry date in the FAITH cohort (date of start of persistent treatment for immune thrombocytopenia).

^†^Index date: date of infection for cases.

Abbreviations: 95% CI: 95% confidence interval; OR: odds ratio.

**Table D.** Full multivariate model* assessing the link between exposure to corticosteroids during the month before index date and occurrence of severe infection. This model had the lowest Akaike criterion value (331.921).

| **Variables** | **OR [95% CI]** | ***p*** |
| --- | --- | --- |
| Male gender | 1.77 [1.20-2.62] | 0.004 |
| Exposure to corticosteroids in the month before index date^†^ | 2.48 [1.61-3.83] | <0.001 |

*Adjusted for mucosal or internal bleeding at diagnosis, lung disease, kidney disease, cardiac disease, diabetes mellitus, exposure to rituximab in the six months before index date, to azathioprine, mycophenolate, cyclosporine and polyvalent immunoglobulin in the month before index date, as well as splenectomy before index date. Age and disease duration until index date were neutralized by matching.

^†^Index date: date of infection for cases.

Abbreviations: 95% CI: 95% confidence interval; OR: odds ratio.

**Table E.** Full multivariate model* assessing the link between exposure to corticosteroids during the month before index date and occurrence of severe infection, without withdrawal from the model of the exposure to rituximab in the month before index date.

| **Variables** | **OR [95% CI]** | ***p*** |
| --- | --- | --- |
| Male gender | 1.78 [1.20-2.63] | 0.004 |
| Exposure to corticosteroids in the month before index date^†^ | 2.57 [1.66-3.97] | <0.001 |
| Rituximab to rituximab in the six months before index date^†^ | 1.67 [0.96-2.90] | <0.07 |

*Adjusted for mucosal or internal bleeding at diagnosis, lung disease, kidney disease, cardiac disease, diabetes mellitus, exposure to rituximab in the six months before index date, to azathioprine, mycophenolate, cyclosporine and polyvalent immunoglobulin in the month before index date, as well as splenectomy before index date. Age and disease duration until index date were neutralized by matching.

^†^Index date: date of infection for cases.

Abbreviations: 95% CI: 95% confidence interval; OR: odds ratio.

**Table F.** Full multivariate model* assessing the link between exposure to corticosteroids during the month before index date and occurrence of severe infection: sensitivity analysis adding the variable corresponding to the occurrence of a hospitalization of at least 7 days between start of follow-up and index date.

| **Variables** | **OR [95% CI]** | ***p*** |
| --- | --- | --- |
| Male gender | 1.67 [1.12-2.48] | 0.012 |
| Exposure to corticosteroids in the month before index date^†^ | 2.59 [1.67-4.05] | <0.0001 |
| Hospitalization of at least 7 days | 2.63 [1.67-4.14] | <0.0001 |

*Adjusted for mucosal or internal bleeding at diagnosis, lung disease, kidney disease, cardiac disease, diabetes mellitus, exposure to rituximab in the six months before index date, to azathioprine, mycophenolate, cyclosporine and polyvalent immunoglobulin in the month before index date, as well as splenectomy before index date. Age and disease duration until index date were neutralized by matching.

^†^Index date: date of infection for cases.

Abbreviations: 95% CI: 95% confidence interval; OR: odds ratio.
